# Retinal nerve fiber layer (RNFL) integrity and its relations to retinal microvasculature and microcirculation in myopic eyes

**DOI:** 10.1186/s40662-018-0120-3

**Published:** 2018-10-10

**Authors:** Dongyi Qu, Ying Lin, Hong Jiang, Yi Shao, Yingying Shi, Shriya Airen, Giovanni Gregori, Jianhua Wang

**Affiliations:** 1 Bascom Palmer Eye Institute, University of Miami Miller School of Medicine, Miami, FL USA; 2 State Key Laboratory of Ophthalmology, Zhongshan Ophthalmic Center, Sun Yat-sen University, Guangzhou, Guangdong China; 3 Department of Neurology, University of Miami Miller School of Medicine, Miami, FL USA; 4 College of Arts and Sciences, University of Miami, Miami, FL USA; 5 Department of Ophthalmology Bascom Palmer Eye Institute, 1638 NW 10th Avenue, McKnight Building - Room 202A, Miami, FL 33136 USA

**Keywords:** Myopia, Retinal nerve fiber layer, Polarization-sensitive optical coherence tomography, Retinal blood flow velocity, Retinal function imager, Retinal microvasculature, Optical coherence tomography angiography

## Abstract

**Background:**

The aim was to determine retinal nerve fiber layer function and its relations to retinal microvasculature and microcirculation in patients with myopia.

**Method:**

Polarization-sensitive optical coherence tomography (PS-OCT) was used to measure phase retardation per unit depth (PR/UD, proportional to the birefringence) of the retinal nerve fiber layer (RNFL). Optical coherence tomography angiography (OCTA) was used to measure macular vessel density analyzed using fractal analysis. In addition, a retinal function imager (RFI) was used to measure macular blood flow velocities in arterioles and venules. Twenty-two patients with moderate myopia (MM, refraction > 3 and < 6 diopters), seventeen patients with high myopia (HM, ≥ 6 D) and 29 healthy control subjects (HC, ≤ 3.00 D) were recruited. One eye of each patient was imaged.

**Results:**

Although the average PR/UD of the RNFL in the HM group did not reach a significant level, the birefringence of the inferior quadrant was significantly lower (*P* < 0.05) in the HM group compared to the HC group. Significant thinning of the average RNFL and focal thinning of RFNL in temporal, superior and inferior quadrants in the HM group were found, compared to the HC group (*P* < 0.05). There were no significant differences of retinal blood flow velocities in arterioles and venules among groups (*P* > 0.05). The macular vessel density in both superficial and deep vascular plexuses was significantly lower in the HM group than in the other two groups (*P* < 0.05) as well as in the MM group than in the HC group (*P* < 0.05). The average PR/UD and PR/UD in the inferior quadrant were not related to refraction, axial length, blood flow velocities and macular vessel densities (r ranged from − 0.09 to 0.19, *P* > 0.05).

**Conclusions:**

The impairment of the retinal nerve fiber birefringence in the HM group may be one of the independent features in high myopic eyes, which appeared not to relate to macular microvascular density and blood flow velocity.

## Background

Myopia is a major public health problem that is growing worldwide [1]. Many studies have investigated structural changes in myopic eyes, mainly the thinning of the peripapillary retinal nerve fiber layer (RNFL) and deformation of the optic nerve head [2–5]. The RNFL thickness of adult myopia is believed to become thinner with an increasing refractive degree of the eyes [3], while others believe that the average RNFL thickness decreases with increasing axial length [6]. Retinal structure changes in high myopia may also be due to thinning of the sclera and retina, retinal vascular narrowing and straightening, retinal hypo-perfusion, choroid and retina microcirculation alteration, increased retinal ischemia and hypoxia, and ganglion cell axonal degeneration [7], all of which may result in the thinning of the RNFL. The anatomic changes in the optic nerve head and surrounding structures in high myopia are becoming more readily evident by OCT imaging. There is altered mechanical stress on the nerve fibers and compromised prelaminar perfusion in myopic eyes. The tilted disc due to high myopia is expected to cause a shift of RNFL entering the optic nerve head. The pattern of RNFL distribution was altered in high myopes with thinner average, superior, nasal, and inferior but thicker temporal nerve fiber layer thickness [8]. In addition to the alterations of the retinal structure, the impairment of the retinal microvascular network was found in myopic eyes [9, 10], while the retinal blood flow velocity remained unchanged [9]. However, the functional changes induced by the structural changes and the possible pathophysiologic mechanisms are still being researched. It is not clear whether the integrity of the RNFL alters in myopic eyes. The aim of this study was to determine retinal nerve fiber layer function and its relations to retinal microvasculature and microcirculation in patients with myopia.

## Methods

The study was approved by the ethics committee board of the University of Miami, and all research subjects were treated in accordance with the tenets of the Declaration of Helsinki. All subjects were recruited and informed about the purposes and methods of the study and each volunteer signed a consent form.

### Study subjects

A total of 22 moderate myopic patients and 17 high myopic patients were recruited at the Bascom Palmer Eye Institute of the University of Miami. According to the refractive diopters, all the eyes were grouped into the moderate myopia (MM) group with refraction more than − 3.00 D and less than − 6.00 D and high myopia (HM) group with refraction of at least − 6.00 D. In addition, 29 healthy control (HC) subjects with refraction less than − 3.00 D were also recruited.

All subjects with following conditions were excluded from the study: 1) any ocular diseases (inflammation, infection and ischemic diseases); 2) any systemic or ocular diseases such as diabetes, hypertension, glaucoma and macular degeneration.

### Study examinations and clinical data analysis

All subjects underwent ophthalmologic examinations, including slit-lamp biomicroscopy, best-corrected visual acuity (BCVA) measurement, fundus examination, and intraocular pressure measurement. Axial length (AL) was measured with the IOL-Master (Zeiss 500, Carl Zeiss Meditec, Inc., Dublin, CA). Refraction data were transformed to spherical equivalent (SE) and calculated as the spherical dioptric power + cylindrical dioptric power. The BCVA was 20/20. Blood pressure, including systolic pressure and diastolic pressure, and heart rate were also measured, and the subjects’ medical and family histories were obtained.

Custom polarization optical coherence tomography (PS-OCT) was used to measure the birefringence of the retinal nerve fiber layer (RNFL). The system was developed based on our existing spectrometer used in our previous studies [11, 12]. Two identical spectrometers were used, and each of them consisted of a diffraction grating (1200 lines/mm), a lens (f = 180 mm) and a line scan camera (Aviiva SM2 CL 2014, Atmel, San Jose, CA). The configuration enabled a scan speed of 24,000 A-scans per second. The light source was a super-luminescent diode (SLD, center wavelength 840 nm with a bandwidth of 50 nm). After vertically polarized, the light was split by a non-polarizing beam splitter into the sample and reference beams. The reference light passed a quarter wave plate (QWP) oriented at 22.5° and was reflected by a mirror. After the double passage of the QWP, the orientation of the polarization plane was set to be 45° to the horizontal meridian, which provided equal reference power in both channels of the two spectrometers. The sample beam passed a QWP oriented at 45°, providing a beam of light of 0.75 mW on the eye, which is well below the safety limit. The light delivery system consisted of a x-y galvanometer scanner with a relay lens (f = 50 mm) and a Volk 60 D ophthalmic lens for scanning the retina. The theoretical axial resolution is 6.2 μm, corresponding to ~ 5 μm in the retina (refractive index of 1.38). Two identical spectrometers were used to acquire polarized light reflected from the sample, and reference aims to detect phase retardation. Thereafter, RNFL birefringence was calculated based on the changes of the retardation versus scan depth in the tissue [13].

**Fig. 1:**
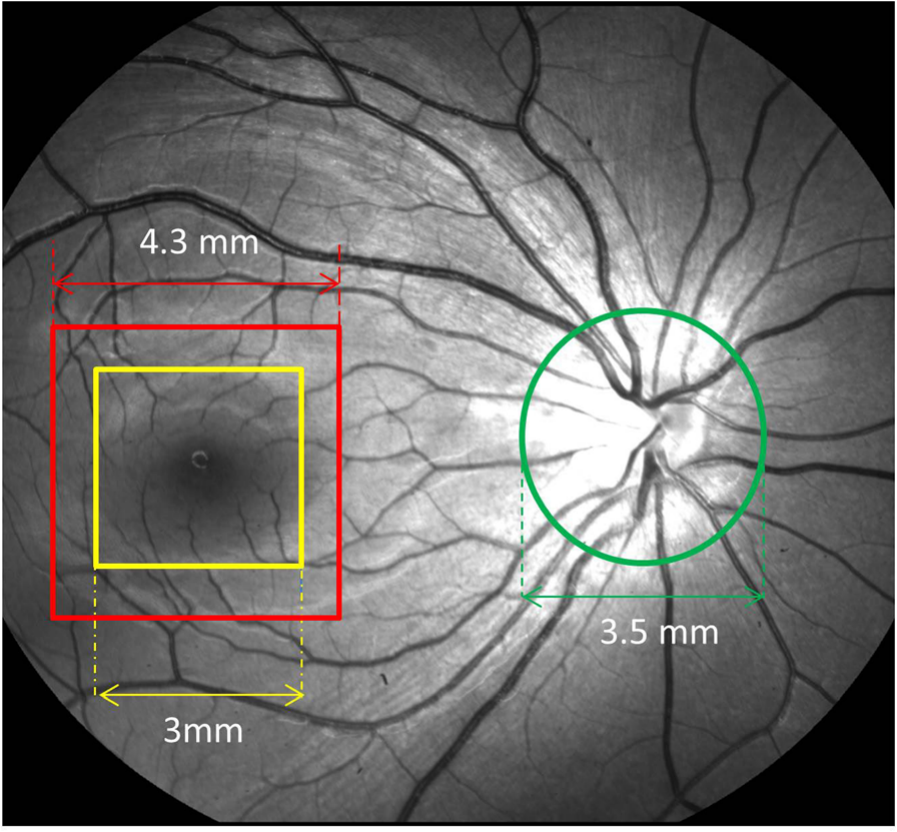
Custom PS-OCT scanning diameter was set to be a 3.5 mm centered on the optic nerve head. OCTA scanning was 3 × 3 mm^2^ centered on the fovea, and the RFI image was taken on an area of 4.3 × 4.3 mm^2^ centered on the fovea

**Fig. 2:**
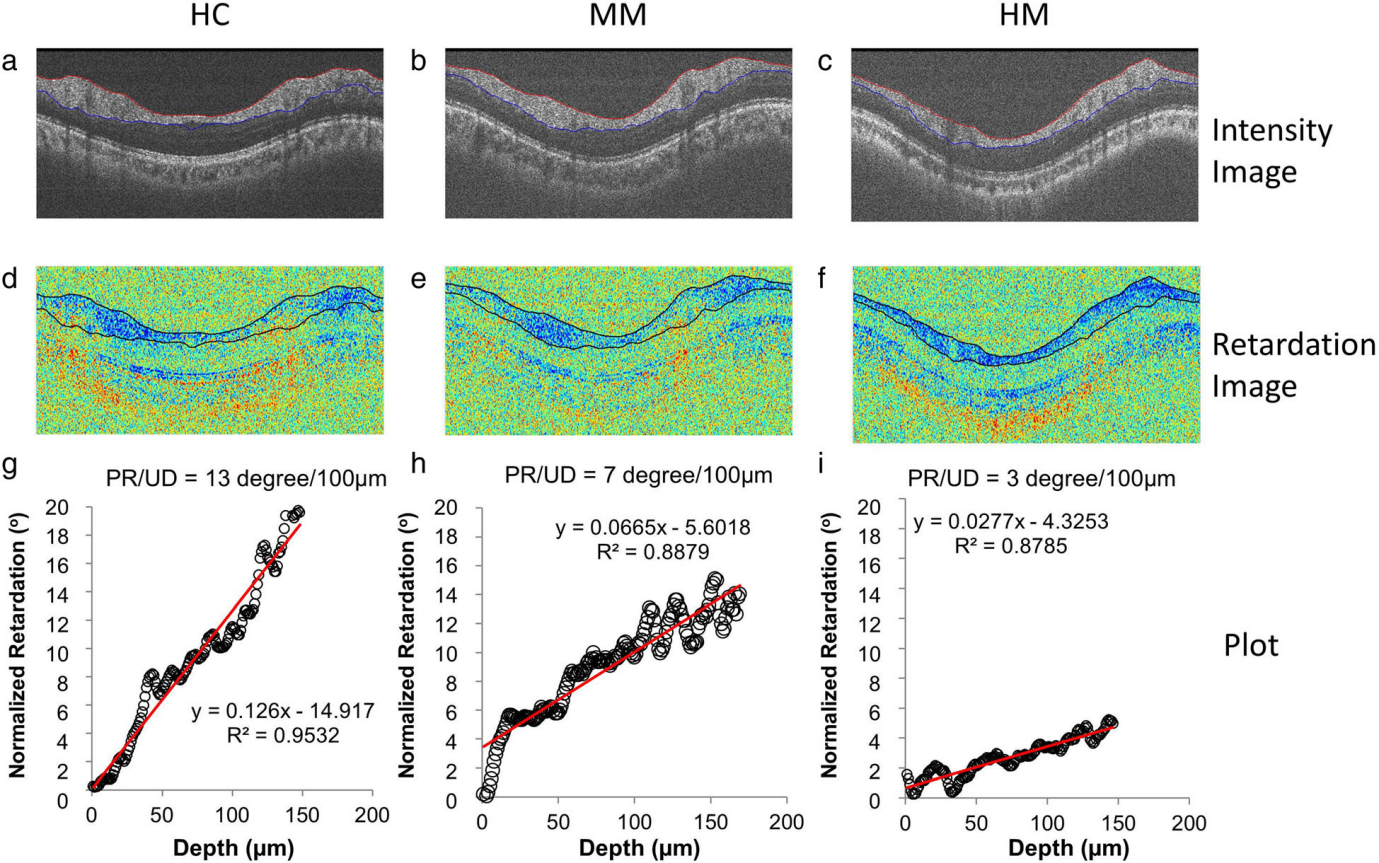
The circumpapillary RNFL imaged using PS-OCT. The OCT intensity images were obtained from a HC subject (**a**), a MM eye (**b**) and a HM eye (**c**). The outlined RNFL boundaries obtained from the OCT intensity images were projected onto the corresponding retardation images (**d-f**). Phase retardations of 512 A-scans in the inferior quadrant were plotted as a function of the scan depth in the RNFL (**g** and **i**) after the signal was normalized. The slopes of the linear regression were defined as the PR/UD (degree/100 μm) of the RNFL, and the results are listed in each panel. HC: healthy control; MM: moderate myopia; and HM: high myopia

During imaging, the subjects sat in front of the device with their chin on the chin rest. The operator adjusted the position and alignment till the eye was fixed on a green dot (fixation target), meanwhile, the center of the optic nerve head was shown on both vertical and horizontal scans. A circle scan was performed around the optic nerve head (3.4 mm in diameter) (Fig. 1). All the raw data were processed using the custom software in Matlab R2008a. The RNFL boundaries were outlined from the OCT intensity images, and the information was then used to extract the phase retardation in the RNFL (Fig. 2). The circular scan contained 2048 A-scans, which were divided into superior, nasal, inferior and temporal quadrants. The phase retardations of each quadrant (512 A-scans) were plotted as a function of the scan depth. For better plotting, signal normalization was done by setting the first pixel of the pRNFL to 0 in the x-axis and the retardation in the first pixel to 0 in the y-axis. This procedure did not alter the slopes obtained by the linear regression. The slopes of the linear regression were defined as the PR/UD (degree/100 μm) of the quadrantal RNFL sections, including the temporal (T), superior (S), nasal (N) and inferior (I) quadrants.

### Optical coherence tomography angiography (OCTA) for measuring macular microvasculature

The retinal microvascular network was imaged using optical coherence tomography angiography (OCTA, Zeiss HD-OCT with AngioPlex, Carl Zeiss Meditec, Dublin, CA). Its technical aspects have been previously described in detail [14]. Briefly, the system is an upgraded version of the Cirrus model 5000, which scans at a rate of 68,000 A-scans per second and non-invasively captures the depth-encoded vasculature in the retina. The angiography is generated by multiple B-scans of the same locations and is analyzed regarding both the intensity and phase information. The algorithm for processing the blood flow information is OCT Microangiographic-Complex (OMAGc), which yields the enface vessel network in different retinal layers. In the present study, a 3 × 3 mm^2^ angiogram was generated in less than 3 s by taking four sequential B-scans in each y-axis location with approximately 9000 B-scans, each being composed of 245 A-scans (A scan = 1 pixel). The OCTA system incorporates a real-time retinal tracking technology named FastTrac™ that ensures the achievement of retinal angiographic images with minimal motion artifacts. Angiograms of the superficial and deep vascular plexus were exported for analysis. The superficial vascular plexus refers to the vascular network from the internal limiting membrane (ILM) to the inner plexiform layer (IPL), while the deep vascular plexus refers to the vascular network from the inner nuclear layer (INL) to the outer plexiform layer (OPL) [15, 16]. The vascular enface view images of these plexuses were exported for further analysis, including magnification correction, separation of large and small vessels and fractal analysis [9]. The magnification for imaging the fundus using fundus photography and the elongation of the eye caused differences of the OCT in the myopic eye. Hence, proper magnification correction was required to evaluate the dimensional information of the retina. The correction is often performed using keratometry measurements or axial length. Bennett’s correction with the axial length provides more accurate data than the keratometry correction method [17]. In the present study, Bennett’s formula [18] was used to determine the scaling factor of the OCT angiograms for the adjustment of the ocular magnification in the moderate and high myopia groups [scaling factor = 3.382 × 0.013062 × (AL-1.82)].

### OCTA data processing

**Fig. 3:**
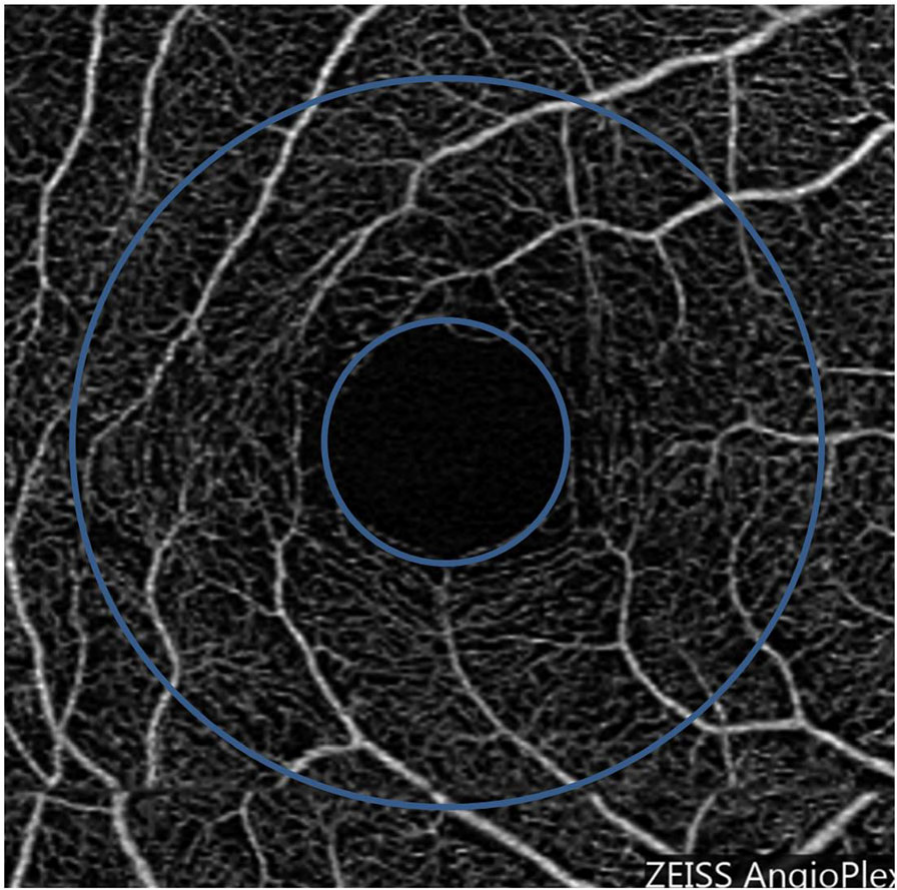
OCTA image. The annulus from 0.6 mm to 2.5 mm was analyzed

Macular scans covered a 3 × 3 mm^2^ area, in which the microvasculature vessel area density was measured (Fig. 3). The macular vessel area density was defined as the fractional area occupied by an annulus of 0.6 mm and 2.5 mm for the inner and outer diameter, respectively. The original OCTA image size was 245 × 245 pixels, which was resized to 1024 × 1024 pixels using the previously described requirements of the vessel segmentation software program [19]. The images of the myopic eyes were then converted to ones with more pixels by using a scaling factor from Bennett’s formula (scaling factor = 3.382 × 0.013062 × (AL-1.82)). The enlarged images were then cropped to a pixel size of 1024 × 1024 pixels. The image mode was greyscaled. The segmentation software, running in the Matlab environment (The Mathworks, Inc., Natick, MA, USA), applied a series of image processing procedures, such as inverting, equalizing and removing background noise and non-vessel structures to create a binary image. Any vessel with a diameter < 25 μm was defined as a microvessel and was separated from the remaining vessels, defined as the large vessels. This procedure first removed the large vessel projection artifacts from the deep vascular plexus [20]. We then skeletonized the images. The center of the foveal avascular zone (FAZ) was recognized by searching the intensity gradient from the image center to the periphery. The image from the left eye was flipped horizontally to match the quadrantal definition and averaging. Fractal analysis was performed in each partition using the box-counting method with the fractal analysis toolbox (TruSoft Benoit Pro 2.0, TruSoft International, Inc., St. Petersburg, FL). According to the default settings in the fractal analysis software, the pixel size of the largest box for counting non-empty boxes was set to 104 pixels, and the incremental rotation degree of the grid in searching non-empty boxes was set to 15 degrees. The fractal dimension (Dbox) was obtained to represent the vessel density in the annulus.

### Retinal function imager for measuring macular blood flow velocity

**Fig. 4:**
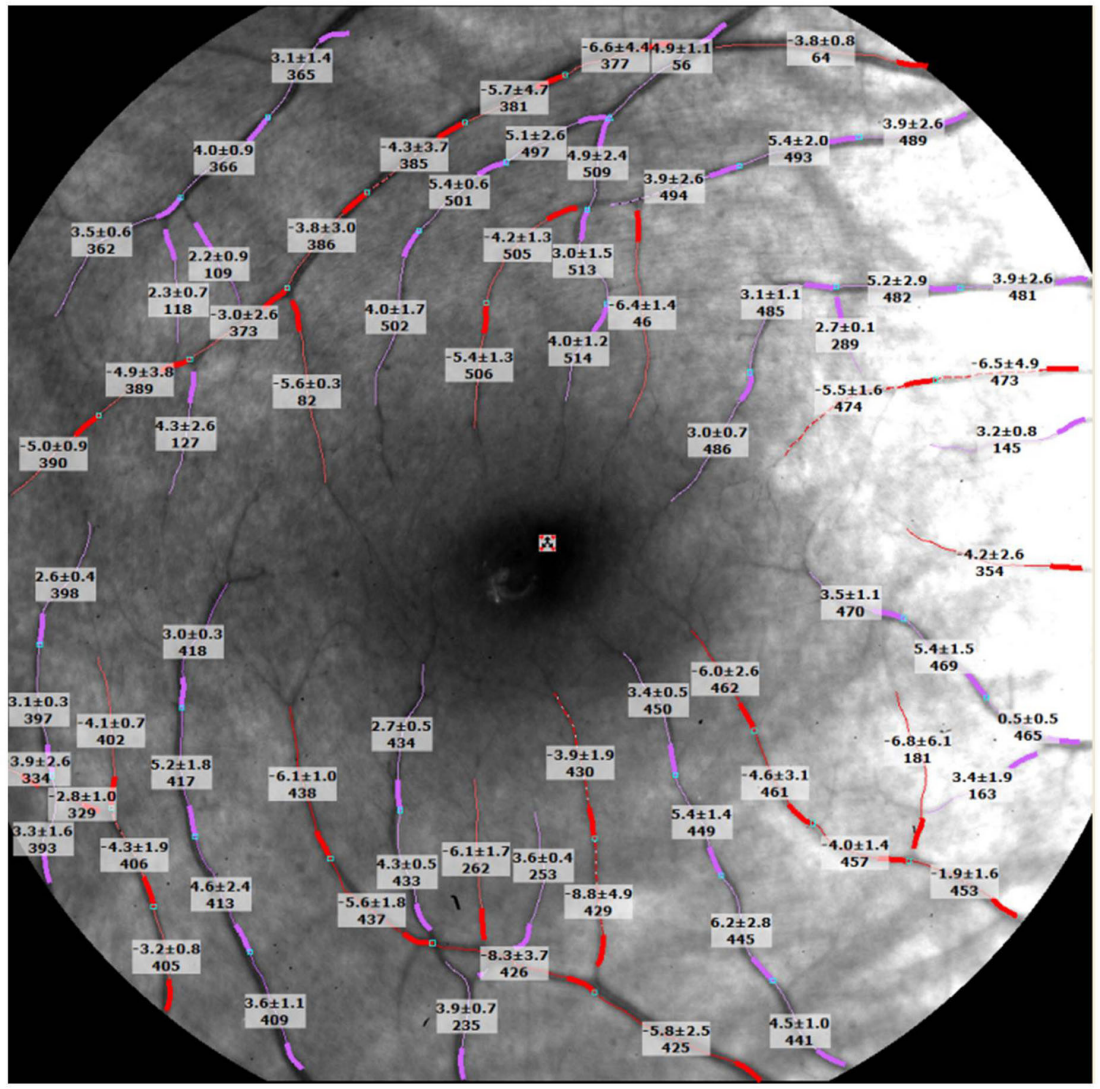
Retinal blood flow velocity measured using the RFI. The retina of a healthy subject imaged using the RFI with a field of view of 20 degrees centered on the fovea (dark area in the center) is shown. The arterioles are marked in red and overlaid with the measured blood flow velocities (expressed as the mean ± standard deviation, vessel identification, unit: mm/s); the venules and their respective velocities are marked in pink. The measured vessels cover the second, third, and fourth branches of the retinal vessels. The negative values represent arteriolar flow towards the fovea, and the positive values represent venular flow away from the fovea

RFI is an advanced ophthalmic multimodal imaging modality, which can directly measure blood flow velocity in small retinal vessels [21–24]. The system has been described previously in detail [21–24]. In brief, a stroboscopic light source and a high-resolution digital camera are used to rapidly take a series of retinal images and yield velocity measurements with its image processing software. The blood flow velocity’s operating mode uses a green (“red-free”) illumination. When the green light penetrates the retina down to the retinal pigment epithelium (RPE), all vessels in the retina above the RPE can be imaged. To control the effect of heart pulsation on flow velocity measurements, a probe is attached to the subject’s finger allowing image acquisition to be synchronized to a selected phase of the patient’s pulse pattern. The digital pictures are captured, stored, and processed by differential imaging, which directly detects moving erythrocytes in retinal vessels as small as 4 μm in width. The measured velocities in the secondary and tertiary branches of the arterioles and venules were also recorded (Fig. 4). RFI imaging does not require any contrast agents [25, 26]. During imaging, 1% tropicamide was used to dilate the pupil, and a well-trained examiner completed this exam. The image field was 4.3 × 4.3 mm^2^ centered on the fovea (RFI setting 20 degrees). In the present study, Bennett’s formula was used to determine a scaling factor, which compensates for the measurement [9].

### Statistical analysis

All data were presented in the format of mean ± standard deviation (SD) and analyzed with the statistical package (Statistic, StatSoft, Inc., Tulsa, OK). One-way analysis of variance (ANOVA) was used, followed by Fisher LSD post hoc test to determine the differences in mean among the three groups. Pearson’s correlation was used to determine the relations among measurements. In addition, the multiple regression was used to determine whether the retinal velocity and vessel density as a whole can predict the pRNFL birefringence. *P* < 0.05 was used as the threshold for statistical significance.

## Results

### Demographics and baseline characteristics

The detailed subjects’ characteristics are summarized in Table 1.

**Table 1:** Demographics and clinical manifestations of study subjects

Variables	HC	MM	HM
Age (years)	30.6 ± 6.6	29.3 ± 5.6	29.5 ± 6.0
	22 – 48	20 – 41	21 – 40
Sex ratio, Male: Female	13 : 16	11 : 11	6 : 11
Spherical equivalent refractive error (D)	−1.39 ± 0.95	−4.63 ± 0.99	−7.28 ± 1.05
Range (D)	0 – −2.75	−3 – −5.75	−6 – −9.25
AL (mm)	24.34 ± 0.72	25.39 ± 1.16*	26.94 ± 0.63*^
SBP (mmHg)	114.97 ± 11.36	113.24 ± 9.09	110.59 ± 11.01
DBP (mmHg)	75.97 ± 8.13	76.67 ± 7.75	71.65 ± 6.43*
HR (beats/min)	69.17 ± 11.69	71.86 ± 7.47	69.82 ± 10.78

Results are presented as the mean ± standard deviation. Abbreviations: *AL* = Axial Length, *D* = diopters, *HC* = healthy control, *HM* = high myopia, *HR* = heart rate, *MM* = moderate myopia, *RE* = refractive error, *SBP* = systolic blood pressure, *DBP* = diastolic blood pressure, *SD* = Standard deviation. *P < 0.05 compared to HC, ^*P* < 0.05 compared to MM

**Fig. 5:**
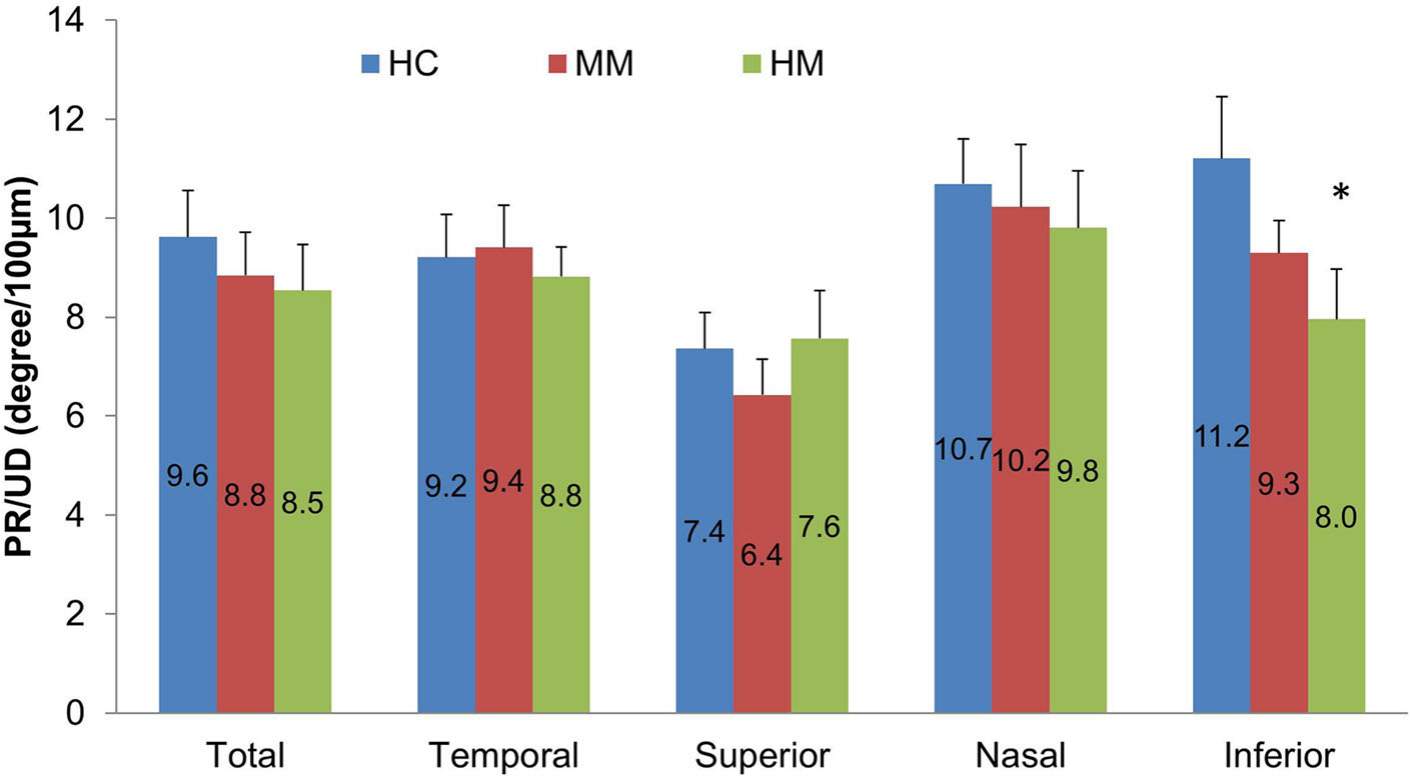
The PR/UD of the RNFL in MM and HM groups compared to the HC group. The birefringence of the inferior quadrant was significantly lower (*P* < 0.05) in the HM group compared to the HC group, although the average birefringence of the RNFL in HM group did not reach significance. Bars = Standard Errors

**Fig. 6:**
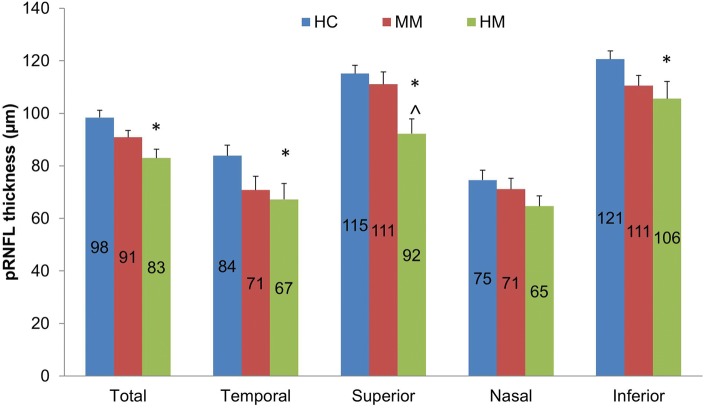
RNFL thickness. Significant thinning of the RNFL was found in the average RFNL and RFNL in the temporal, superior and inferior quadrants of the HM group compared to the HC group (*P < 0.05 compared to HC group, ^*P* < 0.05, compared to MM group). Bars = Standard Errors

**Fig. 7:**
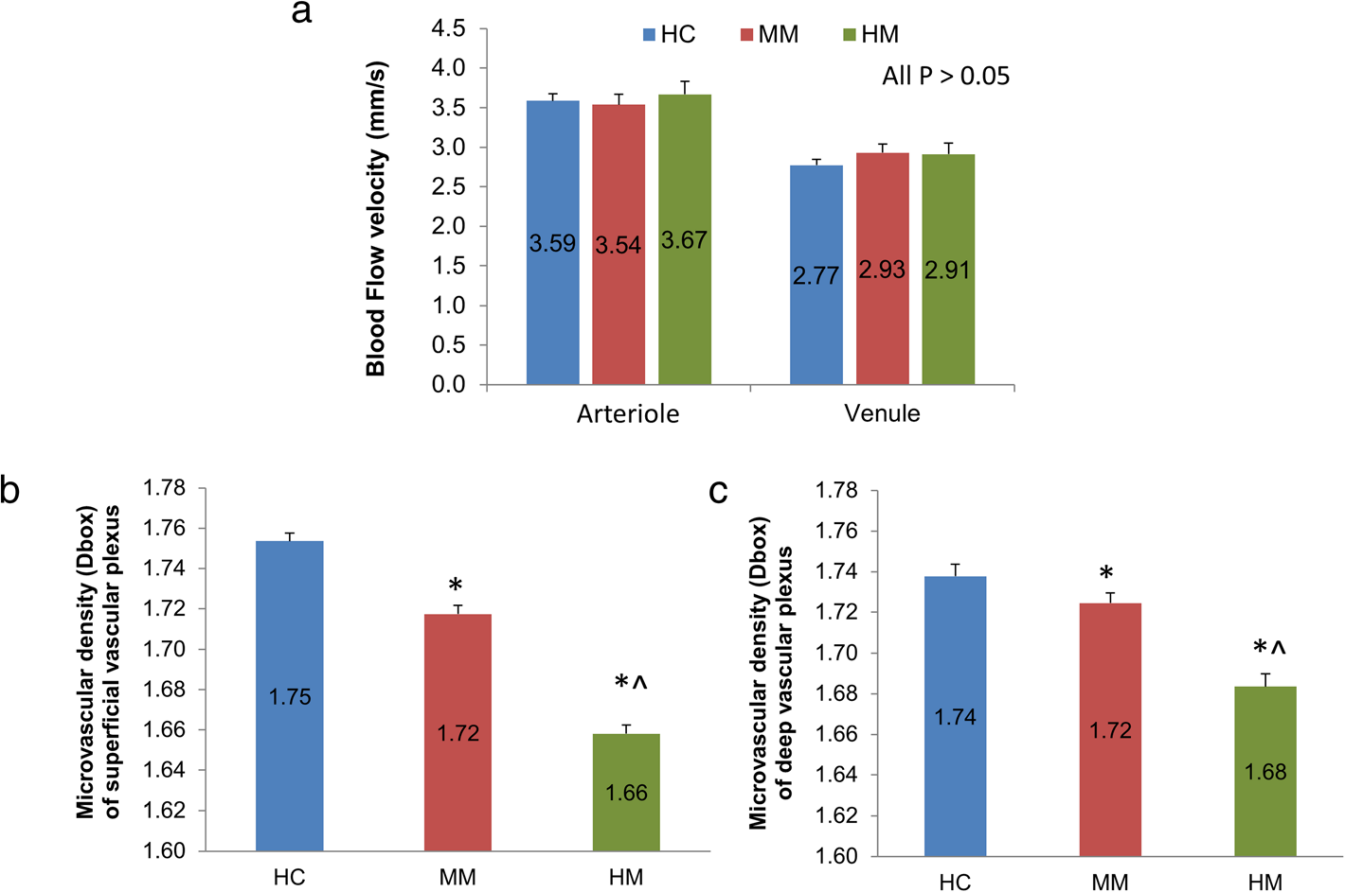
Macular blood flow velocity and microvascular network density. There were no significant differences in retinal blood flow velocities in arterioles and venules among groups (*P* > 0.05). The macular vessel densities in both superficial and deep vascular plexuses were significantly lower in the HM group than in the other two groups (*P* < 0.05) as well as in the MM group than in the HC group (*P* < 0.05). Bars = Standard Errors

**Fig. 8:**
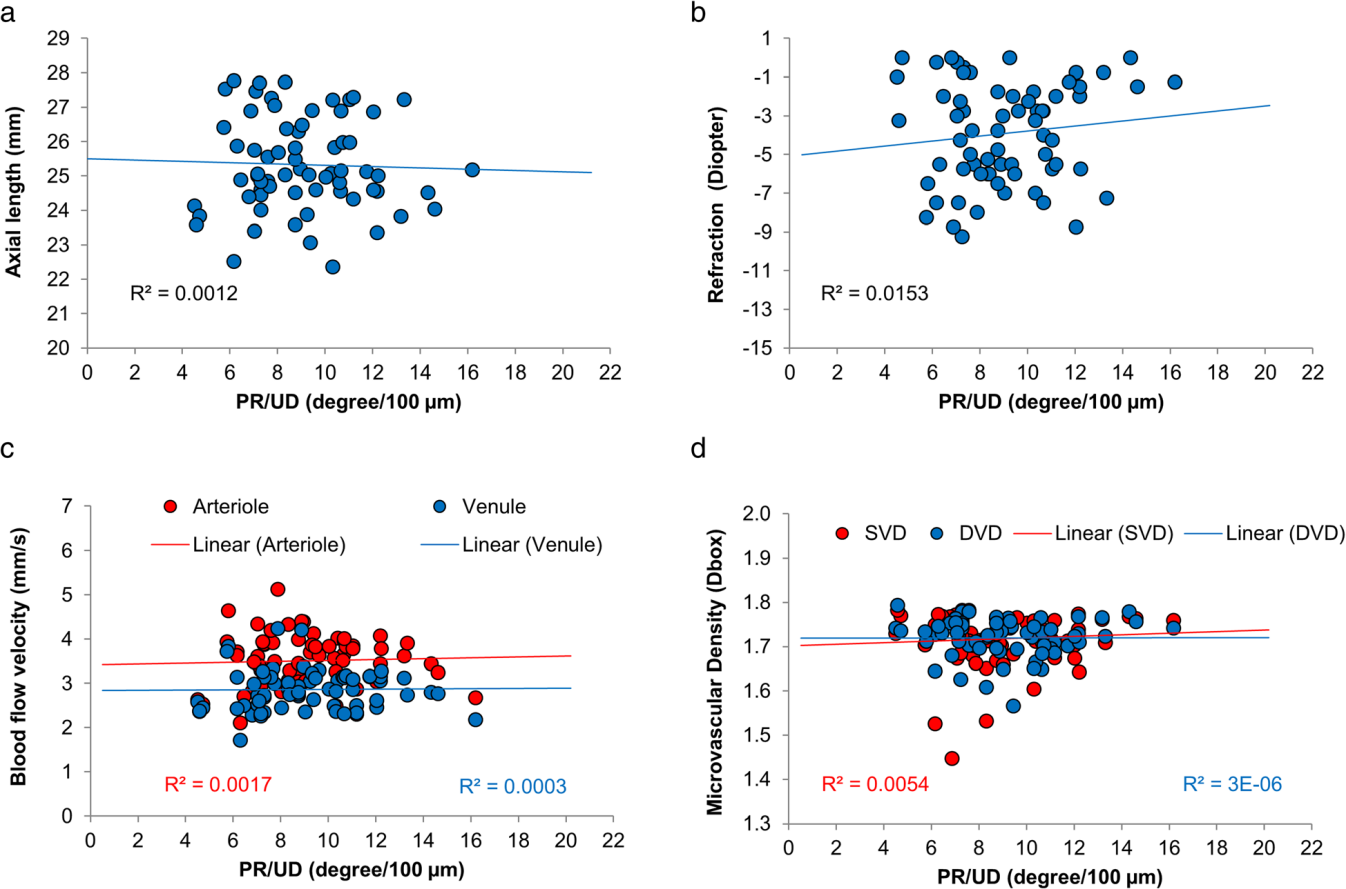
Relations among measurements of RNFL birefringence, macular blood flow velocity, and microvascular density. The average birefringence and birefringence in the inferior quadrant were not related to axial length (**a**), refraction (**b**), blood flow velocities (**c**) and macular vessel densities (**d**) (r ranged from − 0.09 to 0.19, *P* > 0.05). SVD: superficial vascular density; DVD: deep vascular density

Although the average PR/UD of the RNFL in HM group did not reach significance, the birefringence of the inferior quadrant was significantly lower (*P* < 0.05) in the HM group compared to the HC group (Fig. 5). Significant thinning of the RNFL was found in the average RFNL and RFNL in the temporal, superior and inferior quadrants in the HM group compared to the HC group (Fig. 6, *P* < 0.05). There were no significant differences in retinal blood flow velocities in arterioles and venules among groups (Fig. 7, *P* > 0.05). The macular vessel density in both superficial and deep vascular plexuses was significantly lower in the HM group than in the other two groups (*P* < 0.05) as well as in the MM group than in the HC group (*P* < 0.05). The average PR/UD and PR/UD in the inferior quadrant were not related to refraction, axial length, blood flow velocities and macular vessel densities (Fig. 8, r ranged from − 0.09 to 0.19, *P* > 0.05). Multiple regression indicated that the PR/UD was not predictable by refraction, axial length, blood flow velocity and macular vessel densities (F = 0.34, *P* = 0.91).

## Discussion

This study is the first to investigate the RNFL birefringence in myopic eyes and its relations with RNFL thickness, macular microvasculature, and microcirculation. Our results showed significant focal loss of birefringence in the inferior sector of RNFL in subjects with HM compared to the HC, whereas significant decrease of total RNFL thickness, superior and nasal thickness were also observed. It appears that the loss of birefringence was focal and may be secondary to mechanical deformation of the RNFL. This point was further demonstrated by no correlation between birefringence loss and macular microvascular circulation. Furthermore, there was no correlation of inferior birefringence loss with refraction and axial length indicating that the loss of birefringence might be due to the deformation of the optic nerve head, and not simply thinning of the RNFL and elongation of the axial length.

Myopia is one of the most common eye disorders, and the epidemic of high myopia, in particular, is a serious hazard to the vision [27]. It is critical to better understand the function of the RNFL in myopia, in an attempt to develop an imaging biomarker that detects early onset of myopia-related retinopathy. The RNFL birefringence, which arises from the orientation of the neuronal microtubules, allows evaluation of the microtubule and thus cellular integrity [28]. The alteration of the RNFL birefringence of the inferior quadrant in the high myopic eyes found in the present study may indicate that the impairment of the RNFL function co-existed with the thinning of the RNFL and the impairment of retinal microvasculature. Retinal microvasculature was altered in both the MM and HM groups, while the change in the RNFL birefringence was only found in the HM group. This may suggest that the microvascular network impairment may occur prior to the change of birefringence. Further longitudinal studies may reveal which event occurs first.

Interestingly, no relationship was found between the RNFL birefringence and other measurements, including refraction, axial length, macular microvasculature, and microcirculation. This may indicate that the RNFL birefringence is not influenced by eyeball elongation in the early stage of myopia. It could also be speculated that with the elongated eye axis, the macula microvessels would be stretched, eventually resulting in reduced visual function in the later high myopia stage (with myopic retinopathy) [29]. However, the retinal blood flow velocity remains the same, which may help maintain the integrity of the RNFL. This speculation may also explain the thinning of the RNFL possibly due to stretching while the average RNFL birefringence remained unchanged. The cohort of myopic patients in the present study had no detectable retinopathy except for myopia arc and tessellated fundus. Further studies are warranted to determine whether the birefringence of the RNFL alters in myopic eyes with myopic retinal degeneration, which is possibly caused by biomechanical abnormalities such as posterior ectasia.

Through the study of myopia model (chicken) and myopia patients, Shih et al. and Yang et al. [30, 31] found that compared to the control group, the choroidal blood flow in the myopia group decreased, which was related to the refractive index and axial length of the eye. Dimitrova et al. [32] showed that the axial length of myopia patients is closely related to the flow rate of the posterior ciliary artery (PCA) and central retinal artery (CRA), and the flow rates of the PCA and CRA decrease with the increase of the degree of myopia. Benavente-Pérez et al. [33] found that with an increasing degree of myopia, the peak systolic velocity (PSv) and end diastolic velocity (EDv) were all decreased in both outer retinal blood supply short posterior ciliary arteries and the inner retina blood supply central retinal artery. The resistance index (RI) (RI = (PSv-EDv)/PSv) was increased, causing ischemia of the choroid and retina, leading to retinal and choroidal degeneration induced by neovascularization and eventually high myopia fundus changes. Thus, in patients with high myopia, retinal and choroidal degeneration are closely associated with insufficient blood supply to the retinal vascular system and ciliary vascular system, which may be one of the factors of high myopia retinopathy. However, the present study did not find a change of retinal blood flow velocity in myopic eyes.

The combination of a suite of ophthalmic imaging modalities enables the study of a wide range of retina, including RNFL function, structure, microvasculature, and microcirculation. For example, the combination of OCT, OCTA, and RFI was applied to study age-related alterations of retinal structure, microvasculature, and microcirculation [34]. The PS-OCT device was added to the present study to explore the relations between the RNFL birefringence and other measurements. The PS-OCT has been validated and was used to study patients with multiple sclerosis (MS), an inflammatory disease. Impaired RNFL birefringence was found in patients with MS [35]. RFI is an advanced imaging modality which can directly measure blood flow velocity and blood flow, and the system has been used in clinical research with good repeatability [36].

There are limitations to the present study. First, we did not perform a longitudinal study, and therefore, interpretation of the occurrence sequence may be compromised. Although we found only vascular alteration and not birefringence in the MM group, tracking which event occurs first in a longitudinal study will be beneficial. Second, while we found alterations in both vascular and birefringence measurements in high myopic eyes, our sample size is still small, which led to a non-significant difference of the averaged PR/UD (*P* = 0.08). We performed a power calculation based on the measurements using a software program (G*Power, version 3.0.10) developed by Faul [37] and found that we needed to have 87 cases with a detection power of 0.80. As this is the first attempt at quantitative analysis of pRNFL birefringence, the significant alteration of the birefringence in the inferior quadrant may shine some light for future study design. Third, RFI uses visual light (green light). It remains unknown whether the visual light affects transient changes of blood flow velocity. Burgansky-Eliash et al. [38] used RFI and did not find a significant difference between glaucomatous and control eyes. Whether retinal blood flow velocity is altered in glaucomatous eyes compared to control eyes warrants further study; other RFI studies already detected meaningful clinical alterations in diseased eyes. As the only FDA-approved system for measuring retinal blood flow velocity in a large field of view, further validation of the RFI applications may be needed. Fourth, corneal birefringence compensation has been applied in measuring retardation maps of the retina and pRNFL. In a previous study [39], the RNFL surface in the present study was used as a reference in the retardation calculation, so that corneal birefringence may not influence our measurement of PR/UD. Fifth, since the circular scanning pattern was used in the present study to scan the optic nerve head, it is impossible to correct the pRNFL thickness using the Bennett formula. In general, the thickness of the RNFL tends to be smaller in myopic eyes since the diameter of the scan circle is larger. The raster scan pattern would be used in further studies, which could be corrected using the Bennet formula.

## Conclusion

In summary, the impairment of the retinal nerve fiber birefringence in the high myopia group may be one of the independent features in high myopic eyes, which does not seem to relate to macular microvascular density and blood flow velocity.
